# Outcome of Pregnancy-Related Acute Kidney Injury and Resulting Maternal Renal Morbidity in a South Asian Population: A Single-Center Study

**DOI:** 10.7759/cureus.90977

**Published:** 2025-08-25

**Authors:** Rabia Shahid, Adil Manzoor, Ujala Imran, Hafiza Noor Us Sabah, Hajra Amin

**Affiliations:** 1 Nephrology, Pakistan Kidney and Liver Institute and Research Centre, Lahore, PAK

**Keywords:** acute cortical necrosis, acute kidney injury, end stage renal disease (esrd), maternal and fetal outcome, post-partum hemorrhage, pregnancy-related aki, renal biopsy, sepsis

## Abstract

Objective

The study aimed to determine the outcome of pregnancy-related acute kidney injury (PR-AKI) and resulting maternal renal morbidity in a single center of a low-to-middle-income country (LMIC) in the South Asian region.

The objective of the research was to calculate the percentage of patients requiring renal replacement therapy after suffering from PR-AKI, and the reversibility of the insult. A comparison of early versus late presentation after PR-AKI in terms of renal outcome was also made.

Materials and methods

This was a retrospective study conducted in the Nephrology Department of Pakistan Kidney and Liver Institute and Research Centre (PKLI & RC), Lahore, Pakistan. A total of 42 female patients aged 18-45 years presented to the outpatient or acute medical unit of PKLI & RC between April 2018 and April 2025 with PR-AKI. Their clinical course and outcomes were followed for twelve weeks and categorized as resolution versus development of chronic kidney disease (CKD) and/or end-stage renal disease (ESRD). Laboratory investigations, imaging, and renal biopsy findings were tabulated and analyzed using IBM SPSS 27 and Microsoft Excel (Microsoft® Corp., Redmond, WA, USA).

Results

Stage III AKI was most prevalent, seen in 81% (n=34), followed by Stage II AKI in 14.3% (n=6) and Stage I AKI in 4.8% (n=2). The most common cause of PR-AKI was postpartum hemorrhage (PPH), observed in 35.7% (n=15) of women, followed by sepsis in 23.8% (n=10). Other causes included pre-eclampsia, thrombotic microangiopathy (TMA), eclampsia, non-steroidal anti-inflammatory drug (NSAID) abuse, and dehydration.

Early presentation after delivery was associated with better outcomes in terms of maternal renal morbidity and progression to ESRD. Patients who progressed to ESRD presented at an average of 43.5 ± 20.6 days after delivery, whereas those who did not progress presented earlier, at an average of 27.9 ± 29.6 days. This difference in presentation days according to ESRD status was statistically significant (p = 0.009). No statistically significant correlation was found between pregnancy-induced hypertension (PIH), eclampsia, or pre-eclampsia and progression to CKD and/or ESRD (p > 0.05). In terms of maternal renal outcome, CKD was diagnosed in 66.6% (n=28), of which 42.8% (n=18) became dialysis dependent. Complete resolution of PR-AKI was observed in 28.5% (n=12). Maternal mortality was observed in 16.2% (n=6), with 9.5% (n=4) being dialysis dependent at the time of death. Causes of maternal mortality ranged from septic shock to multi-organ failure in the background of acute kidney injury. Fetal mortality was observed in 30.6% (n=11), including both intrauterine and neonatal deaths. Pre-eclampsia was found to be significantly associated with fetal mortality (p = 0.023), with the odds of fetal mortality due to pre-eclampsia being 13.71 (1.31-143.44) times higher than in non-pre-eclampsia patients.

Conclusion

Early presentation and timely intervention are crucial for preserving maternal renal function after PR-AKI. Best practices should be implemented, especially in poverty-stricken areas, to avoid complications such as PPH and sepsis that may lead to AKI in pregnant or peri-partum women. It is imperative to strengthen antenatal screening, skilled birth attendance, and early referral systems to minimize the burden of avoidable complications.

## Introduction

Living in a low-income setting has a significant impact on healthcare due to lack of infrastructure and accessibility. Pregnant women constitute a high-risk population and are susceptible to numerous complications and morbidities during pregnancy. A myriad of complications can occur during the antepartum, peripartum, and postpartum phases. Regular antenatal care is critical to ensure that risks are identified early and timely interventions are made for optimal fetal and maternal outcomes [1].

Various complications during pregnancy have been associated with an increased risk of chronic illness in the mother later in life. A meta-analysis conducted on 44,165 women of South Asian origin in 2021 by Gadve SS et al. showed that women with gestational diabetes mellitus were at 10.81 times higher risk of developing type 2 diabetes mellitus than those without gestational diabetes [2]. Pregnancy-induced hypertension, gestational diabetes, eclampsia, pre-eclampsia, placental abruption, preterm labor, stillbirth, and pregnancy loss have all been linked with a higher risk of developing hypertension, type II diabetes (T2DM), and cardiovascular disease later in life [3].

Acute kidney injury (AKI) refers to the sudden decline in kidney function due to an insult. This decline can be quantified by a reduction in glomerular filtration rate (GFR), an increase in serum creatinine, or a drop in urine output. Previously known as acute renal failure, AKI is often reversible, either completely or partially, in most cases. Causes of AKI can be categorized into pre-renal, intra-renal, or post-renal etiologies. However, these are not always distinct, and some overlap is expected [4].

Pregnancy has been identified as a state of physiological stress for the female body. The growing fetus, fluctuating hormones, and the ultimate act of childbirth can all impact the mother’s homeostasis, leading to possible complications for both mother and fetus. Pregnancy-related acute kidney injury (PR-AKI) is one such complication. There is a paucity of local data on the subject; however, a study conducted in 2022 in a low-resource setting reported a prevalence rate of 68 cases of PR-AKI per 100,000 births [5]. Lack of awareness, especially in rural areas, remains a significant issue, as many people still opt for home births or fall prey to unqualified practitioners in Pakistan [6]. The Pakistan Demographic and Health Survey reported that 28.8% of Pakistani women delivered at home with the assistance of unqualified traditional birth attendants between 2015 and 2019 [7]. Unsanitary conditions and unskilled attendants predispose mothers to life-threatening complications such as sepsis, postpartum hemorrhage, and perineal trauma [8]. AKI in such patients may be caused by volume depletion from postpartum hemorrhage or by acute tubular necrosis (ATN) resulting from sepsis.

There is no universally accepted specialized tool or criterion specific to PR-AKI for diagnosing and managing its complexity [9]. Different studies have used varying criteria. For the purpose of this study, the KDIGO (Kidney Disease: Improving Global Outcomes) guidelines for AKI were applied [10]. Serum creatinine was the only marker used in this retrospective study. A potential limitation of this methodology was the exclusion of urine output from the definition of AKI. Non-standardization of urine output estimation makes it an unreliable tool for AKI staging. However, it is important to acknowledge that excluding this criterion entirely could have led to missed or underdiagnosed cases. This study was designed to correlate international data with local findings and compare the burden of disease. According to a meta-analysis that included studies conducted between 1980 and 2021, Trakarnvanich T et al. reported the global incidence of PR-AKI to be 2% [11]. It is important to note the stark contrast between developed and low-to-middle-income countries (LMICs). The incidence of PR-AKI in LMICs has been reported to range between 4-26%, whereas in high-income countries it varies from 1-2.8% [5].

## Materials and methods

Study design and objectives

This was a single-center retrospective study designed to determine the outcome of PR-AKI and resulting maternal renal morbidity.

Primary Outcome

Renal outcomes of patients after PR-AKI in terms of complete resolution of AKI, partial resolution of AKI (progression to CKD), or ESRD, i.e., dialysis dependence at 12 weeks after the incidence of AKI.

Staging was done according to KDIGO guidelines [10].

Secondary Outcomes

The secondary outcomes included correlation of early versus delayed presentation with outcomes of PR-AKI, assessment of maternal and fetal mortality in patients with PR-AKI, and evaluation of the association of PIH, pre-eclampsia, eclampsia, and Hemolysis, Elevated Liver enzymes, and Low Platelets (HELLP) syndrome with progression to ESRD, maternal mortality, and fetal mortality.

Study duration

The study was conducted from April 2018 to April 2025.

Study setting

The study was conducted at the Pakistan Kidney and Liver Institute and Research Center (PKLI & RC) in Lahore, Punjab, Pakistan. The center is a quaternary care facility specializing in diseases of the liver and kidneys, including transplantation and hemodialysis as modalities of kidney replacement therapy (KRT). The hospital caters only to registered patients who fall under its scope of services. The referral system does not include purely obstetric cases, as there is no in-house obstetrical unit.

Study population

The study included all women diagnosed with PR-AKI in the OPD or acute medical unit (AMU) of PKLI & RC, Lahore during the study period.

Inclusion Criteria

All women diagnosed with PR-AKI presenting to PKLI & RC, Lahore during the study period.

Exclusion Criteria

Women with any other etiology of renal failure/acute kidney injury and patients with incomplete medical records or those lost to follow-up before three months of PR-AKI were excluded.

A total of 50 women were selected by non-probability consecutive sampling. Of these, 8 were excluded due to incomplete records or lack of follow-up visits to accurately chart the outcome of AKI. Ultimately, 42 patients were included in the study. They represented both urban and rural populations.

Data collection and procedure

Data were collected from the electronic medical records maintained in Sisoft Healthcare Information Systems (SisoHbys Version: 2.0.4.495) for each patient. Additional information was supplemented using manual medical records. Extracted data included patient age, date of presentation, mode of presentation (OPD versus AMU), postpartum days (to compare early versus late presentation), and biopsy findings. In this study, only serum creatinine values were used as a marker of renal function. The cause and outcome of PR-AKI were noted for every patient. Maternal renal outcome was defined as complete resolution of AKI, progression to CKD, or progression to ESRD at 12 weeks after diagnosis of AKI. Twelve weeks (three months) was taken as the cut-off for defining CKD. Fetal mortality was also recorded. Each patient’s record was reviewed for the presence of PIH, pre-eclampsia, eclampsia, and HELLP syndrome to assess statistically significant correlations with the incidence and outcome of PR-AKI.

Data were validated by trained medical doctors at every stage.

Operational definitions

AKI

KDIGO criteria were used to define AKI, which is an increase in serum creatinine to 1.5-1.9 times baseline values within 7 days, or an absolute increase of ≥0.3 mg/dL. KDIGO guidelines also include urine output <0.5 mL/kg/h for more than 6 hours as a diagnostic criterion for AKI. However, urine output was not accounted for in this study, and only serum creatinine values were used.

Pregnancy-related AKI (PR-AKI): An abrupt decline in kidney function during pregnancy up to twelve weeks postpartum [12].

Stages of AKI:* *KDIGO guidelines were used for staging, applying only the serum creatinine criterion for diagnosis [10].

Stage I AKI was defined as 1.5-1.9 times increase in serum creatinine from baseline or an absolute increase of ≥0.3 mg/dL.

Stage II AKI was defined as 2.0-2.9 times increase in serum creatinine from baseline.

Stage III AKI was defined as ≥3 times increase in serum creatinine from baseline, an absolute value >4 mg/dL, or initiation of dialysis.

Complete resolution of AKI: Serum creatinine level returned to baseline at or before twelve weeks of follow-up from first presentation.

Partial resolution of AKI: Serum creatinine level did not return to baseline, but dialysis was not required within twelve weeks of first presentation.

Progression to CKD

Persistently impaired kidney function with either structural or functional abnormality, where the GFR remained between 15 and 59 mL/min/1.73 m² at three months after AKI diagnosis, without continued dependence on dialysis [13].

ESRD

Continuous need for renal replacement therapy in the form of dialysis or kidney transplant, denoting irreversible loss of kidney function [14].

Data analysis

The collected data were verified against manual records. Complete datasets were entered and analyzed using IBM SPSS 27 and Microsoft Excel (Microsoft® Corp., Redmond, WA, USA).

Numerical data such as age and postpartum day of presentation were presented as mean ± SD. Categorical data, including cause and stage of AKI, fetal and maternal mortality, and renal outcomes, were presented as frequencies and percentages.

Differences in postpartum days of presentation according to ESRD status were determined using the Mann-Whitney U test. Fisher’s exact test was applied to determine associations between PIH and outcomes. A p-value ≤0.05 was considered statistically significant.

Ethical clearance

The study was conducted after obtaining approval from the Institutional Review Board (IRB) of PKLI & RC, Lahore (IRB number: 00522024). Patient profiles and data were anonymized, and confidentiality was ensured throughout the study. The IRB waived the requirement for informed consent due to the retrospective nature of the study design.

## Results

Demographics

A total of 50 patients selected via non-probability consecutive sampling were enrolled in the study. Of these, 8 patients were excluded because they were either lost to follow-up or had incomplete medical records. Ultimately, 42 female patients diagnosed with PR-AKI and presenting to PKLI & RC, Lahore during the study period fulfilled the inclusion criteria. They represented both urban and rural populations. The mean age was 28.2 years with a standard deviation of ±5.4 years. The mode of presentation was OPD for 41 patients (97.6%), while 1 patient (2.4%) presented directly to the AMU. Among the 42 patients, 30 (71.4%) were primigravidas (nulliparous) and 12 (28.6%) were multiparous.

Renal biopsy

Renal biopsy was performed in 40.4% (n=17) of patients. Echogenic kidneys in 2 patients (4.7%) contraindicated biopsy. Biopsy findings showed acute tubular necrosis (ATN) in 23.5% (n=4) and cortical infarction in 23.5% (n=4). Acute cortical necrosis and thrombotic microangiopathy (TMA) were each reported in 17.6% (n=3). Severe acute and chronic interstitial nephritis with tubular injury was seen in 2 patients (4.7%). One patient (2.4%) had an insufficient sample, and a conclusive study could not be performed. Renal biopsy is considered the gold standard for establishing a confirmed diagnosis in PR-AKI. However, it is not always possible to perform one. Contraindications include overt sepsis/infection, pyelonephritis, bleeding disorders or deranged coagulation profile, uncontrolled hypertension, shock, and marked echogenicity of the kidneys. In some cases, patients or their families may also refuse consent for the procedure. The findings from this study are likely applicable to the broader population, as there were no particular differences between the biopsy subgroup and the overall cohort. No selection bias was observed.

Stage of PR-AKI

**Figure 1:**
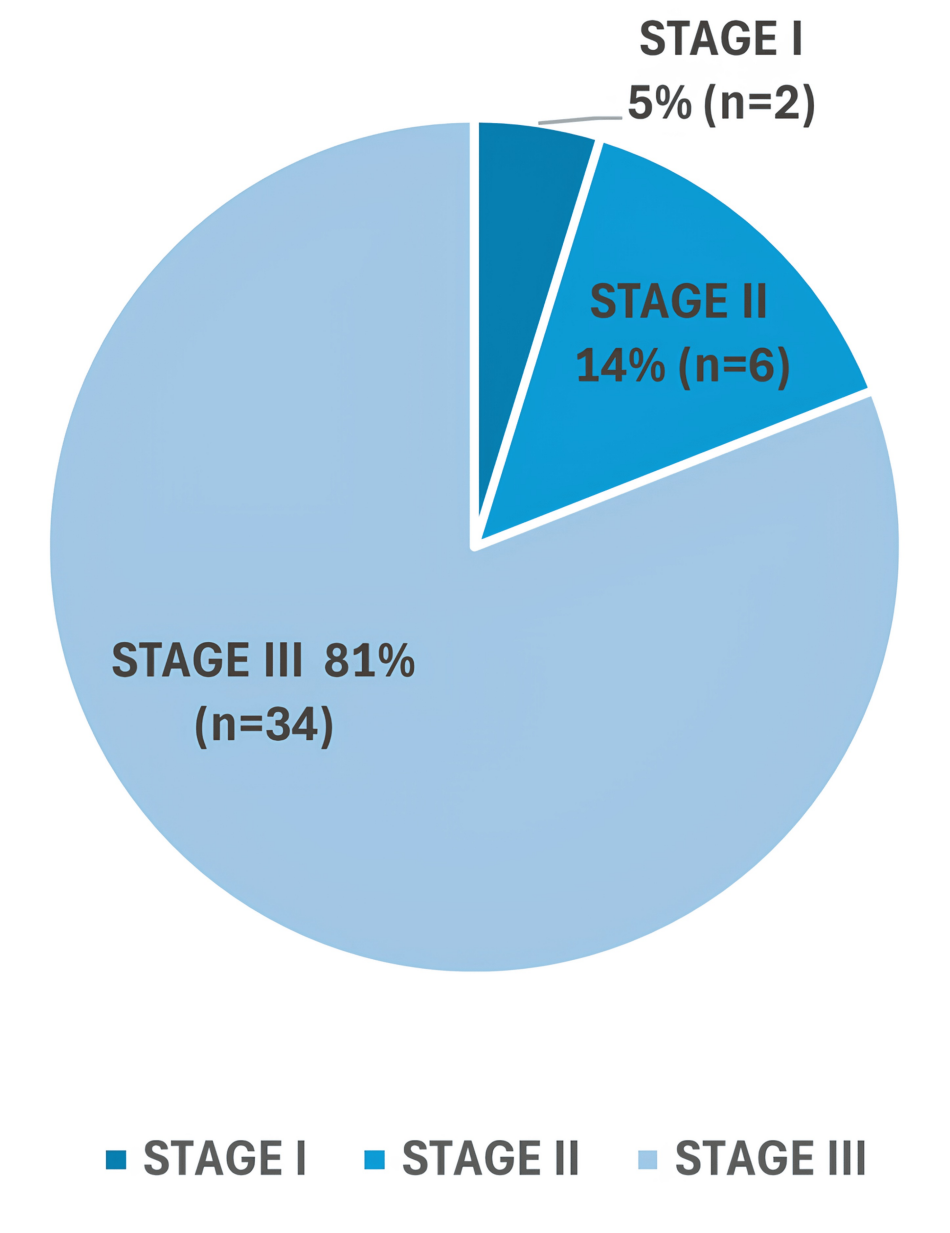
Frequency distribution according to stage of AKI. Stages of pregnancy-related acute kidney injury (PR-AKI) as per KDIGO guidelines [10]. Data are presented as percentages and n, where n = number of patients.

Stage III AKI was the most prevalent, seen in 81% (n=34), followed by Stage II AKI in 14.3% (n=6). Only 4.8% (n=2) presented with Stage I PR-AKI. The frequency distribution of PR-AKI based on staging is illustrated in Figure 1.

Causative factors of PR-AKI

**Figure 2:**
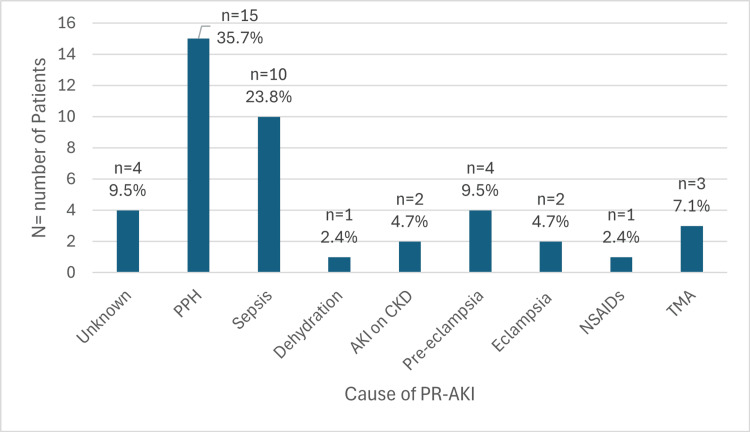
Frequency distribution according to cause of pregnancy-related acute kidney injury (PR-AKI). Data are presented as n (%), where n is the number of patients/cases according to the various causes of PR-AKI and % indicates the frequency distribution of each cause. PPH: Postpartum hemorrhage; AKI: Acute kidney injury; CKD: Chronic kidney disease; NSAIDs: Non-steroidal anti-inflammatory drugs; TMA: Thrombotic microangiopathy.

Given the limitations of the retrospective study design and lack of multivariate analysis, the exact cause of PR-AKI could not be definitively established. However, causative factors were explored. The most common cause of PR-AKI was postpartum hemorrhage (PPH), seen in 35.7% (n=15) of women, consistent with findings from similar studies in the region. This was followed by sepsis-related PR-AKI in 23.8% (n=10). Other causes included pre-eclampsia in 9.5% (n=4), TMA in 7.1% (n=3), eclampsia in 4.8% (n=2), non-steroidal anti-inflammatory drug (NSAID) abuse in 2.4% (n=1), and dehydration in 2.4% (n=1). The cause of PR-AKI was unknown in 9.5% (n=4). Pre-existing CKD was identified in 4.7% (n=2) of patients, both of whom presented with an acute insult due to ongoing UTI. The distribution of causative factors is shown in Figure 2.

Outcome of AKI

Early presentation after delivery was associated with better outcomes in terms of maternal renal morbidity and progression to ESRD. The difference in the number of postpartum days at presentation according to ESRD status was determined by the Mann-Whitney U test. A p-value ≤0.05 was considered significant. Patients who progressed to ESRD presented at an average of 43.5 ± 20.6 days after delivery, whereas patients who did not progress presented earlier, at an average of 27.9 ± 29.6 days. This difference in presentation days according to ESRD status was statistically significant (p = 0.009).

The mean age of ESRD patients was 25.6 ± 3.3 years, while the mean age of patients without ESRD was 31.5 ± 5.5 years. This difference was statistically significant (p < 0.001).

No statistically significant correlation was found between PIH, eclampsia, or pre-eclampsia with progression to CKD and/or ESRD (p > 0.05), as shown in Table 1.

**Table 1:** Correlation of CKD and ESRD with pre-eclampsia, PIH, eclampsia, and HELLP in PR-AKI. CKD: Chronic kidney disease; ESRD: End-stage renal disease; PIH: Pregnancy-induced hypertension; HELLP: Hemolysis, Elevated Liver enzymes, and Low Platelets. Fisher’s exact test was used. Data are presented as n (%), where n = number of patients. A p-value ≤ 0.05 was considered statistically significant.

	Category	CKD		p-value	ESRD		p-value
		Yes n=25 (69.4%)	No n=11 (30.6%)		Yes n=18 (48.6%)	No n=19 (51.4%)	
Pre-eclampsia	Yes n=6 (16.7%)	4 (16.0%)	2 (18.2%)	>0.999	3 (16.7%)	3 (15.8%)	>0.999
	No n=30 (83.3%)	21 (84.0%)	9 (81.8%)		15 (83.3%)	16 (84.2%)	
Pregnancy-induced hypertension (PIH)	Yes n=5 (13.9%)	3 (12.0%)	2 (18.2%)	0.631	2 (11.1%)	3 (15.8%)	>0.999
	No n=31 (86.1%)	22 (88.0%)	9 (81.8%)		16 (88.9%)	16 (84.2%)	
Eclampsia	Yes n=3 (8.3%)	2 (8.0%)	1 (9.1%)	>0.999	2 (11.1%)	1 (5.3%)	>0.999
	No n=33 (91.7%)	23 (92.0%)	10 (90.9%)		16 (88.9%)	18 (94.7%)	
HELLP	Yes n=1 (2.8%)	1 (4.0%)	0 (0.0%)	>0.999	1 (5.6%)	0 (0.0%)	0.486
	No n=35 (97.2%)	24 (96.0%)	11 (100.0%)		17 (94.4%)	19 (100.0%)	

Table 2 shows the association of fetal and maternal mortality with pre-eclampsia, eclampsia, HELLP syndrome, and PIH. Among patients with fetal mortality, pre-eclampsia was present in 4 (36.4%), PIH in 6 (18.2%), and eclampsia in 2 (18.2%); HELLP syndrome was not observed in any patient. Pre-eclampsia was found to be significantly associated with fetal mortality (p = 0.023). The odds of fetal mortality due to pre-eclampsia were 13.71 (1.31-143.44) times higher than in non-pre-eclampsia patients.

**Table 2:** Association of fetal and maternal mortality with PIH, pre-eclampsia, eclampsia, and HELLP in patients with PR-AKI. PIH: Pregnancy-induced hypertension; HELLP: Hemolysis, elevated liver enzymes, and low platelets; PR-AKI: Pregnancy-related acute kidney injury. Fisher’s exact test was used. Data are presented as n (%), where n = number of patients. A p-value ≤ 0.05 was considered statistically significant.

Status	Categories	Fetal mortality		p-value	Maternal mortality		p-value
		Yes n=11(30.6%)	No n=25 (69.4%)		Yes n=6 (16.2%)	No n=31 (83.8%)	
Pre-eclampsia	Yes n=5 (13.9%)	4 (36.4%)	1 (4.0%)	0.023	0 (0.0%)	5 (16.1%)	0.567
	No n=31 (86.1%)	7 (63.6%)	24 (96.0%)		6 (100.0%)	26 (83.9%)	
Pregnancy-induced hypertension (PIH)	Yes n=6 (16.7%)	2 (18.2%)	4 (16.0%)	1	2 (33.3%)	4 (12.9%)	0.245
	No n=30 (83.3%)	9 (81.8%)	21 (84.0%)		4 (66.7%)	27 (87.1%)	
Eclampsia	Yes n=3 (8.3%)	2 (18.2%)	1 (4.0%)	0.216	0 (0.0%)	3 (9.7%)	>0.999
	No n=33 (91.7%)	9 (81.8%)	24 (96.0%)		6 (100.0%)	28 (90.3%)	
HELLP	Yes n=1 (2.8%)	0 (0.0%)	1 (4.0%)	1	0 (0.0%)	1 (3.2%)	>0.999
	No n=35 (97.2%)	11 (100.0%)	24 (96.0%)		6 (100.0%)	30 (96.8%)	

Maternal mortality, on the other hand, was not associated with any of these pregnancy-related conditions.

In terms of maternal renal outcome, CKD was diagnosed in 66.6% (n=28), of which 42.8% (n=18) progressed to ESRD and became dialysis dependent. Complete resolution of PR-AKI was observed in 28.5% (n=12). Two patients (4.7%) expired during the acute phase of presentation, before their renal outcome could be determined. Maternal mortality was observed in 16.2% (n=6), of which 9.5% (n=4) were dialysis dependent at the time of death. Fetal mortality was observed in 30.6% (n=11) cases.

**Figure 3:**
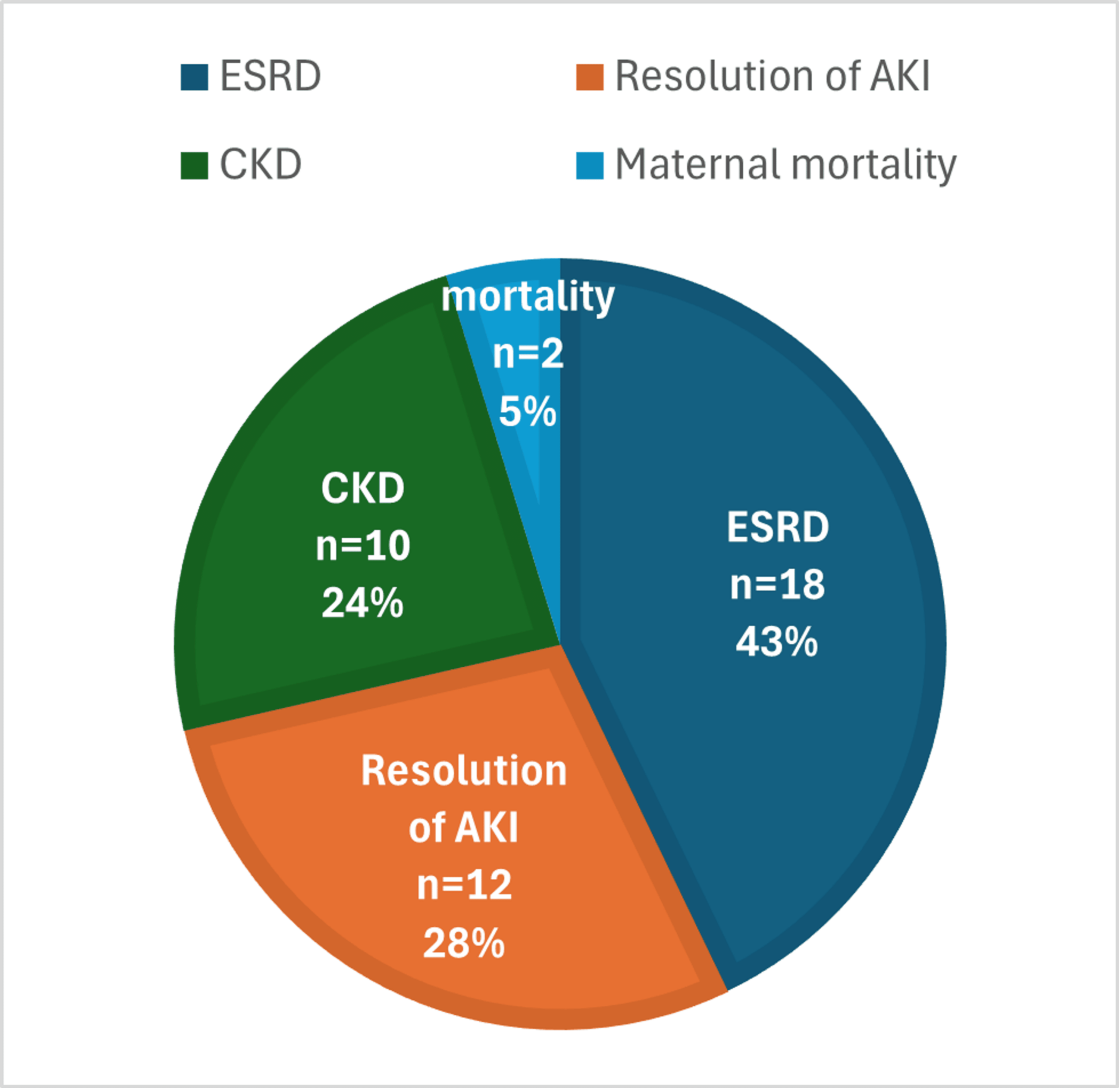
Frequency distribution of maternal outcome after PR-AKI. Data are presented as n (%), where n is the number of patients with the specified outcome. CKD: Chronic kidney disease; ESRD: End-stage renal disease; PR-AKI: Pregnancy-Related Acute Kidney Injury.

The distribution of PR-AKI outcomes is illustrated in Figure 3.

Kidney replacement therapy (KRT) was initiated in 79% (n=33) of patients. The number of dialysis sessions varied from 2 to 23 among patients in whom PR-AKI resolved. A total of 18 (42.8%) patients were labeled as ESRD with irreversible kidney function loss and became dialysis dependent.

## Discussion

Pregnancy-related complications are often difficult to diagnose due to the normal physiological changes that occur during gestation [15]. Increased blood flow to the kidneys during pregnancy elevates the GFR [16], which indirectly decreases serum creatinine levels. According to a systematic review on serum creatinine in pregnancy, Wiles K et al. reported that mean serum creatinine values during pregnancy were 84%, 77%, and 80% of nonpregnant mean values in the first, second, and third trimesters, respectively [17]. The normal range of serum creatinine in pregnancy is therefore lower, creating a risk that PR-AKI may be underdiagnosed or missed [18]. This study was conducted in a quaternary care hospital specializing in liver and kidney diseases, so the true burden of disease could not be fully assessed. As there was no in-house obstetrical unit, all patients were referrals from various healthcare facilities. Of the 42 patients with PR-AKI, complete resolution was observed in 12 (28%). In this study, 66.6% (n=28) of patients had varying degrees of CKD at three-month follow-up after PR-AKI, and 42.8% (n=18) of these patients became dialysis dependent, requiring a renal transplant in the future. This proportion of persistent renal failure is significantly higher than reported in similar studies. For example, a study conducted in a tertiary hospital in Pakistan found that only 14% of patients had persistent renal failure at twelve weeks postpartum [19]. This difference is likely due to the latter being conducted in a teaching hospital with a high-volume gynecology and obstetrics department, where prompt diagnosis and timely management were possible. By contrast, our study only included women who had delivered elsewhere and were later referred for PR-AKI management. The statistically significant difference between early and late presentation in this study further supports the argument that timely diagnosis and intervention are critical to prevent irreversible kidney damage. Lack of awareness and delayed presentation to specialized centers were key reasons why women from lower socio-economic backgrounds were more likely to become dialysis dependent [20]. Considering the retrospective study design and absence of multivariate analysis, causation cannot be firmly established; however, several causative factors identified in our research were consistent with findings from similar studies.

PPH is a well-recognized cause of PR-AKI due to volume depletion leading to reduced renal perfusion. It was the most prevalent cause of PR-AKI in this study. Sudden blood loss can result in hypoperfusion of vital organs, including the kidneys, leading to hypoxia and subsequent infarction, as indicated by cortical infarcts and cortical necrosis observed in the renal biopsies of affected patients. Hemorrhagic or hypovolemic shock caused by PPH may thus lead to irreversible kidney damage from cortical necrosis. PPH was observed in 35.7% of patients in our study, consistent with findings from Haroon F et al., where 36.9% of PR-AKI cases were due to PPH [21]. Similarly, Mir MM et al. reported that PPH accounted for 25% of PR-AKI cases in Jammu and Kashmir, India, between 2013 and 2015 [22]. Uterine atony has been identified as the most common cause of PPH in a meta-analysis by Yunas I et al. [23]. The high prevalence of PPH in this population can be attributed to inadequate healthcare facilities. Even in areas with available resources, home births attended by unqualified local birth attendants are often chosen over hospital-based deliveries, further increasing risks. There is a pressing need for educational campaigns and national awareness programs to ensure that every pregnant woman can make an informed decision. Additionally, timely and systematic referral systems must be established to enable prompt management of complications.

Other potential causative factors of PR-AKI include trauma, retained placental tissue, and coagulation disorders. It is imperative to educate the public on the importance of antenatal care and adherence to the recommended number of healthcare visits during pregnancy. Timely diagnosis remains crucial to avoiding long-term complications.

Sepsis is another important contributing factor, accounting for 23.8% of cases in this study. Mahesh E et al. reported that sepsis contributed to PR-AKI in 59% of patients [24]. A prospective study on pregnant and postpartum females admitted with sepsis showed that 44% of these patients developed PR-AKI [25]. An unhygienic birthing environment, failure to follow standardized aseptic techniques, and lack of proper sterilization equipment all contribute to obstetrical sepsis. The burden of disease can be significantly reduced by adhering to standard infection prevention and control strategies.

Hypertensive disorders of pregnancy, including PIH, pre-eclampsia, eclampsia, and HELLP, are also important contributors to the development of PR-AKI. While hemorrhage and sepsis may be more prevalent in developing nations, these disorders pose a significant healthcare burden in developed countries as well. There has been a reported rise in PR-AKI in the United States and Canada in recent years [26]. In Canada, the incidence of PR-AKI increased from 1.6 per 10,000 deliveries in 2003 to 2.3 per 10,000 in 2007 [26]. Similarly, in the United States, PR-AKI rates rose from 2.3 to 4.5 per 10,000 deliveries between 1998 and 2008 [27]. It has been hypothesized that evolving management approaches for pre-eclampsia and eclampsia may be contributing to this increase in renal failure during pregnancy [28]. In LMICs, delayed diagnosis and late presentation remain the primary drivers of severe morbidity in women with hypertensive disorders of pregnancy [29]. Hypertension is often diagnosed only in hypertensive crises, reflecting a lack of routine monitoring. General awareness about regular physical examinations is limited, and cultural or religious barriers often prevent women from having their blood pressure checked, especially when paramedics are male. Home blood pressure monitoring is also frequently inadequate or unreliable in such households.

Renal biopsy remains the gold standard for diagnosing renal impairment caused by intrinsic parenchymal disease or tubular injury [30]. Tissue diagnosis allows identification of microscopic injury and distinction between important causes of PR-AKI. However, pre-renal causes can typically be identified through clinical presentation and response to interventions such as fluid resuscitation. Renal biopsy is not always feasible in resource-limited settings due to financial constraints. Moreover, pregnancy is a hypercoagulable state, and invasive procedures such as biopsy carry risks, including thrombosis. Biopsies after 28 weeks are generally avoided to minimize risks such as sepsis, bleeding, hematoma formation, or the need for interventions (e.g., embolization or nephrectomy) in case of hemorrhage. Such complications can also precipitate preterm labor.

Limitations

The limitations of this study include its relatively small sample size, which undermines the ability to assess the true burden of disease. As the study was conducted in a transplant facility specializing in liver and kidney care, it is likely that only a limited number of cases were captured. The absence of an in-house obstetrical unit further restricted accurate assessment of PR-AKI incidence. Diagnosing PR-AKI is inherently challenging because creatinine levels in pregnancy are lower than normal, increasing the likelihood that cases were missed due to apparently normal values. Only serum creatinine was used for diagnosis and follow-up of PR-AKI, and urine output was not quantified. In many cases, baseline creatinine values were unavailable, making it difficult to accurately assess complete recovery. Multivariate analysis was not performed, and patient comorbidities were not considered. Consequently, full causality could not be established in this retrospective single-center study. Limitations in controlling for confounding variables, as well as the inability to demonstrate temporality, further restricted causal inference.

Renal biopsy could only be performed in 40% of patients, which might have aided in establishing definitive diagnoses in cases of intrarenal injury. Additionally, poor follow-up in LMICs and inadequate antenatal care mean that many women may have had undiagnosed CKD or chronic glomerulonephritis prior to conception.

## Conclusions

The study highlights the grave prognosis for women who present late with PR-AKI. Timely management remains the cornerstone for the resolution of AKI, irrespective of pregnancy status. Risk factors for PR-AKI, such as hypertensive disorders and dehydration, can be reduced through regular antenatal follow-up. Adherence to aseptic techniques during delivery, ensuring adequate hydration, minimizing blood loss, and timely referral to specialized healthcare centers in the event of complications can help reduce the burden of PR-AKI.

The complication of PR-AKI represents a significant public health challenge in developing nations. Our study concludes that early diagnosis, intervention, and management are critical in minimizing maternal renal morbidity and feto-maternal mortality. The urgent need is to establish nationwide protocols for reporting PR-AKI, particularly from basic and rural healthcare centers, where many cases arise but often go undocumented or lack follow-up. Mass awareness campaigns should be launched to educate the public, healthcare workers, and birth attendants about the risks and preventive measures.

Future studies should be conducted in large-volume tertiary care centers with well-established in-house obstetrics and nephrology services to accurately assess the true burden of PR-AKI. A national registry must be developed, and proper training provided to all healthcare personnel involved. Local birth attendants and Lady Health Visitors (LHVs) at basic health units should be mobilized and educated to play a pivotal role in early detection and referral. Ultimately, timely diagnosis and prompt referral to specialized healthcare facilities can significantly improve outcomes of PR-AKI in low-income settings.
